# Granulocyte-Colony Stimulating Factor-Overexpressing Mesenchymal Stem Cells Exhibit Enhanced Immunomodulatory Actions Through the Recruitment of Suppressor Cells in Experimental Chagas Disease Cardiomyopathy

**DOI:** 10.3389/fimmu.2018.01449

**Published:** 2018-06-25

**Authors:** Daniela N. Silva, Bruno S. F. Souza, Juliana F. Vasconcelos, Carine M. Azevedo, Clarissa X. R. Valim, Bruno D. Paredes, Vinicius P. C. Rocha, Gisele B. Carvalho, Pamela S. Daltro, Simone G. Macambira, Carolina K. V. Nonaka, Ricardo Ribeiro-dos-Santos, Milena B. P. Soares

**Affiliations:** ^1^Center for Biotechnology and Cell Therapy, Hospital São Rafael, Salvador, Brazil; ^2^Gonçalo Moniz Institute, FIOCRUZ, Salvador, Brazil; ^3^National Institute of Science and Technology for Regenerative Medicine, Rio de Janeiro, Brazil; ^4^Federal University of Bahia (UFBA), Salvador, Brazil

**Keywords:** mesenchymal stem cells, granulocyte-colony stimulating factor, immunomodulation, Chagas disease, cardiomyopathy

## Abstract

Genetic modification of mesenchymal stem cells (MSCs) is a promising strategy to improve their therapeutic effects. Granulocyte-colony stimulating factor (G-CSF) is a growth factor widely used in the clinical practice with known regenerative and immunomodulatory actions, including the mobilization of regulatory T cells (Tregs) and myeloid-derived suppressor cells (MDSCs). Here we evaluated the therapeutic potential of MSCs overexpressing G-CSF (MSC_G-CSF) in a model of inflammatory cardiomyopathy due to chronic Chagas disease. C57BL/6 mice were treated with wild-type MSCs, MSC_G-CSF, or vehicle (saline) 6 months after infection with *Trypanosoma cruzi*. Transplantation of MSC_G-CSF caused an increase in the number of circulating leukocytes compared to wild-type MSCs. Moreover, G-CSF overexpression caused an increase in migration capacity of MSCs to the hearts of infected mice. Transplantation of either MSCs or MSC_G-CSF improved exercise capacity, when compared to saline-treated chagasic mice. MSC_G-CSF mice, however, were more potent than MSCs in reducing the number of infiltrating leukocytes and fibrosis in the heart. Similarly, MSC_G-CSF-treated mice presented significantly lower levels of inflammatory mediators, such as IFNγ, TNFα, and Tbet, with increased IL-10 production. A marked increase in the percentage of Tregs and MDSCs in the hearts of infected mice was seen after administration of MSC_G-CSF, but not MSCs. Moreover, Tregs were positive for IL-10 in the hearts of *T. cruzi*-infected mice. *In vitro* analysis showed that recombinant hG-CSF and conditioned medium of MSC_G-CSF, but not wild-type MSCs, induce chemoattraction of MDSCs in a transwell assay. Finally, MDSCs purified from hearts of MSC_G-CSF transplanted mice inhibited the proliferation of activated splenocytes in a co-culture assay. Our results demonstrate that G-CSF overexpression by MSCs potentiates their immunomodulatory effects in our model of Chagas disease and suggest that mobilization of suppressor cell populations such as Tregs and MDSCs as a promising strategy for the treatment of chronic Chagas disease. Finally, our results reinforce the therapeutic potential of genetic modification of MSCs, aiming at increasing their paracrine actions.

## Introduction

Mesenchymal stem cells (MSCs) are known to participate in tissue homeostasis and repair processes in different physiological and pathological settings (1). MSCs have the ability to migrate to injury sites and promote tissue repair through the secretion of trophic and immunomodulatory factors (2). Currently, there is great interest in investigating the therapeutic potential of MSCs, since they are easily obtainable, expandable, and can act by modulating the microenvironment through the secretion of soluble factors, with systemic repercussions (3). The results of clinical trials with MSC-based therapies, however, have frequently been heterogeneous, possibly due to product and patient heterogeneity, but also to the complexity involved in the administration of living cells that respond differently to different microenvironments, leading to unpredictable outcomes (4). Therefore, the development of strategies that can increase and optimize the paracrine actions of MSCs to enhance the effectiveness of MSC-based therapies is highly desired.

The use of genetically modified MSCs, aiming to deliver bioactive factors systemically or locally to damaged tissues may improve their therapeutic potential (5). Granulocyte-colony stimulating factor (G-CSF) has received significant attention in the regenerative medicine field due to well-known actions, especially regarding the mobilization of bone marrow-derived stem cells to the peripheral blood (6). Moreover, G-CSF exerts direct regenerative actions, including protective effects over cardiomyocytes, which express the G-CSF receptor (7). Recently, G-CSF has also been described as a tumor-derived factor that recruits and expand myeloid-derived suppressor cells (MDSCs), which secrete cytokines involved in the induction of regulatory T cells (Tregs), contributing to the immunosuppressive tumor microenvironment (8). These mechanisms of immune escape are now being applied to potentiate immunomodulatory interventions to treat inflammatory diseases (9).

Chagas disease cardiomyopathy, caused by chronic infection with *Trypanosoma cruzi*, is characterized by chronic myocardial inflammation and fibrosis due to parasite persistence and inflammation, that may ultimately lead to chronic heart failure (10). Treatment with MSCs was previously shown to be effective in promoting immunomodulation in the experimental model of Chagas disease (11, 12). After treatment with MSCs, increased levels of anti-inflammatory cytokines, such as TGF-β or IL-10, were induced in mice chronically infected with *T*. *cruzi* (11–13). Moreover, we have previously described that treatment with G-CSF in the mouse model of Chagas disease cardiomyopathy is associated with mobilization of Tregs and modulation of cardiac inflammation and fibrosis (14).

Due to its beneficial properties and different mechanisms of actions of G-CSF and MSCs, we hypothesized that G-CSF-overexpressing MSCs (MSC_G-CSF) present increased therapeutic actions in chronic Chagas disease, through the synergistic association of MSCs’ paracrine actions with the effects of local release of G-CSF in the myocardium. Therefore, in this study we investigated the therapeutic potential of MSC_G-CSF in a mouse model of chronic Chagas disease, and evaluated the participation of suppressor cells in the control of this inflammation-driven cardiomyopathy.

## Materials and Methods

### Animals

Six- to eight-week-old female C57BL/6 mice were used for *T. cruzi* infection or to evaluate the number of leukocytes in the peripheral blood. Male GFP transgenic C57BL/6 mice were used for harvest of bone marrow cells and splenocytes. All animals were raised and maintained in the animal facility of the Center for Biotechnology and Cell Therapy, Hospital São Rafael (Salvador, Brazil), and provided with rodent diet and water *ad libitum*. Animals were handled according to the NIH guidelines for animal experimentation. All procedures described had prior approval from the local animal ethics committee under number 012/09 (FIOCRUZ, Bahia, Brazil).

### Cultures of MSCs

Wild-type bone marrow-derived MSCs were obtained from male GFP transgenic C57BL/6 mice. A genetically modified MSC line with stable overexpression of hG-CSF (MSC_G-CSF) was previously generated and characterized by our group (15). MSCs were cultured in Dulbecco’s Modified Eagle’s Medium (DMEM) supplemented with 10% fetal bovine serum and 1% penicillin/streptomycin (ThermoFisher Scientific, Waltham, MA, USA) in a humidified incubator at 37°C and 5% CO_2_, with complete medium replacement every 3 days. In order to validate dose, route of administration and to assess *in vivo* biological activity of the G-CSF overexpressing MSCs, naïve C57BL/6 mice, were intraperitoneally injected with the cell suspensions, and peripheral blood was collected for 7 days for leukocyte counts. Control group was treated with vehicle (saline), under the same conditions. Mice were anesthetized with inhaled isoflurane (Abbott, Chicago, IL, USA), allowing for peripheral blood to be collected by tail vein puncture. The number of leukocytes was determined by analysis in a hematological counter BC 3000 Plus (Mindray, Shenzhen, China).

### *T. cruzi* Infection and Cell Therapy

Trypomastigotes of the myotropic Colombian *T. cruzi* strain were obtained from culture supernatants of infected LLC-MK2 cells. C57BL/6 mice were infected by intraperitoneal injection with 1,000 *T. cruzi* trypomastigotes in 100 µL PBS. Six months after the infection, mice were randomly assigned into three groups for administrations of MSCs, MSC_G-CSF, or saline. Age-matched naïve mice were used as normal controls. Cell transplantation was performed by weekly intraperitoneal injections of cell suspensions containing 10^6^ MSCs or MSC_G-CSF. An equal volume of vehicle (100 µL) was used in the saline group. At different time points, mice were euthanized by cervical dislocation, under anesthesia with ketamine (100 mg/kg) and xylazine (10 mg/kg). Depending on the time point evaluated, *T cruzi*-infected mice received one (7 days-time point), four (30 days-time point), or seven cell administrations (60 days-time point). In the latter, the fifth administration of MSCs or MSC_G-CSF was performed by close-chest echography-guided intramyocardial injection, as previously described (11). Four independent experiments were performed.

### Flow Cytometry Analysis and Cell Sorting

Cell suspensions were obtained from digested cardiac tissue samples of infected mice treated with MSCs, MSC_G-CSF, or saline, as previously described (16). The cell suspensions were allowed to pass through a 70 µm cell strainer (BD Biosciences, Franklin Lakes, NJ, USA) and counted with a hematocytometer. Aliquots of 10^6^ cells were used for each test tube and 1 µL of Fc blocking reagent (BD Biosciences) was added. Fluorochrome-conjugated antibodies used were: CD11b-PE-Cy5, CD45-APC and GR-1-FITC, or Ly6C-FITC and Ly6C-PE (BD Biosciences). Samples were incubated with the antibodies for 20 min at RT. Sample acquisition was performed using a BD LSRFortessa SORP cytometer using BD FacsDiva v.6.2. Acquired data were analyzed by FlowJo v7.5 (FlowJo Enterprise, Ashland, OR, USA). CD11b^+^GR-1^+^ MDSCs were sorted from digested hearts of MSC_G-CSF-treated mice or from the bone marrow, as indicated in Section “Results.” The cells were stained with GR-1-PE (BD Biosciences), CD11b-APC (ThermoFisher Scientific), and CD45-APC-Cy7 (BD Biosciences), using a FACS Aria cell sorter (BD Biosciences), achieving a purity of approximately 98%.

### Immunofluorescence Analyses

Frozen heart sections of 10 µm were fixed with 4% paraformaldehyde and incubated overnight at 4°C with the following primary antibodies: anti-Foxp3 (Santa Cruz Biotechnology, Dallas, TX, USA) and anti-CD3 (BD Biosciences) or anti-IL10 (BD Biosciences) diluted 1:1,500, 1:200, and 1:50, respectively. On the following day, the sections were incubated for 1 h with secondary antibodies anti-goat IgG Alexa fluor 488-conjugated and anti-rat IgG Alexa fluor 594-conjugated diluted 1:200 (Thermo Scientific). Nuclei were stained with 4,6-diamidino-2-phenylindole (VectaShield mounting medium with DAPI H-1200; Vector Laboratories, Burlingame, CA, USA). The presence of fluorescent cells was determined by observation A1^+^ confocal microscope (Nikon, Tokyo, Japan). Quantifications of CD3/FoxP3^+^ cells were performed in 10 random fields captured under 400× magnification, using the Image Pro Plus v.7.0 software (Media Cybernetics, Rockville, MD, USA).

### MDSCs Migration Assay

Bone marrow cells were obtained from tibiae and femurs of C57BL/6 mice. Mononuclear cells were then isolated by Ficoll–Paque gradient (GE healthcare, Boston, MA, USA) and washed with PBS twice. MDSCs (CD11b/GR-1^+^) were obtained by FACS. Migration was evaluated using a modified 3 μM-pore size Boyden chamber (Cell Biolabs, San Diego, CA, USA) with the lower chamber filled with serum-free conditioned media obtained from MSCs or MSC_G-CSF, and the upper chamber filled with 10^6^ MDSCs. DMEM (Life technologies) or DMEM supplemented with 50 µg/mL Filgrastim (Aché, Guarulhos, Brazil) were used as controls. After 4 h, the cells in the bottom wells were counted using a hemocytometer. The experiments were performed in biological triplicates.

### Lymphocyte Proliferation Assay

Splenocytes (10^6^ cells/well) obtained from EGFP mice were plated in 96-well plate and stimulated with concanavalin A (Con A; 2 µg/mL, Sigma-Aldrich, St. Louis, MO, USA). Purified MDSCs sorted from the hearts of infected mice treated with MSC_GCSF were co-cultured in a 1:10 ratio with Con A-stimulated splenocytes. After incubation at 37°C and 5% CO_2_ for 72 h, cell proliferation was measured as the number of GFP^+^ proliferative blast cells for treated-cells in comparison to untreated cells. Dexamethasone was used as a positive control. Image acquisition and quantification of blasts were performed using the Operetta High Content Imaging System (Perkin Elmer, Waltham, MA, USA). Images were segmented using the Find Cells building block of the Harmony 3.5.2 software (Perkin Elmer), which provides a dedicated algorithm for segmenting digital phase-contrast images. GFP^+^ proliferative blast cells were tracked using the Track Objects building block. The experiments were performed three times.

### Functional Analyses

Animals were anesthetized by isoflurane inhaled (0.5%) to obtain the Electrocardiography records performed using the Bio Amp PowerLab System (PowerLab 2/20; ADInstruments, Sydney, NSW, Australia), recording the bipolar lead I. All data were acquired for computer analysis using Chart 5 for Windows (PowerLab). Records were bandpass filtered (1 to 100 Hz) to minimize environmental signal disturbances. The sampling rate was 1 kHz. The ECG analysis included heart rate and arrhythmias. Treadmill test was performed 6 months after *T. cruzi* infection, as a baseline evaluation, and 8 months after infection (60 days after the treatment). A motor-driven treadmill chamber for one animal (LE 8700; Panlab, Barcelona, Spain) was used to exercise the animals. The speed of the treadmill and the intensity of the shock (mA) were controlled by a potentiometer (LE 8700 treadmill control; Panlab). Room air was pumped into the chamber at a controlled flow rate (700 mL/min) by a chamber air supplier (Oxylet LE 400; Panlab). The mean room temperature was maintained at 21 ± 1°C. After an adaptation period of 20 min in the treadmill chamber, the mice exercised at five different velocities (7.2, 14.4, 21.6, 28.8, and 36.0 m/min), with increasing velocity after 10 min of exercise at a given speed. Total running time was recorded. Velocity was increased until the animal could no longer sustain a given speed and remained 10 s on an electrified stainless-steel grid, which provided an electrical stimulus to keep the mice running.

### Morphometric Analyses

Groups of mice were euthanized 60 days after the therapy under anesthesia, 5% ketamine, and 2% xylazine, the hearts were removed and fixed in 10% buffered formalin. Heart sections were analyzed by light microscopy after paraffin embedding, followed by standard hematoxylin and eosin (H&E) staining. Inflammatory cells were counted using the software Image Pro Plus v.7.0 (Media Cybernetics). The number of inflammatory cells was determined by counting 10 fields (400× magnification) per heart section. Sirius red-stained sections were entirely digitalized using a confocal microscope A1^+^ (Nikon). The percentage of fibrosis was determined by analysis of whole sections stained with Sirius red-stained heart sections and semiautomatic morphometric quantification using Image Pro Plus v.7.0. Two blinded investigators performed the analyses.

### Reverse Transcription Quantitative PCR (RT-qPCR)

Total RNA was isolated from heart samples using TRIzol reagent (Thermo Scientific) and concentration was determined by photometric measurement. High Capacity cDNA Reverse Transcription Kit (Thermo Scientific) was used to synthesize cDNA of 1 µg RNA following manufacturer’s recommendations. RT-qPCR assays were performed to detect the expression levels of *Tbet* (Mm00450960_m1), *Gata3* (Mm00484683_m1), *Tnf* (Mm00443258_m1), *Ifng* (Mm00801778_m1), *Nos2* (Mm01309898m1), *Arg1* (Mm00475988_m1) *Il6* (Mm0446190_m1), and *Il10* (Mm00439616_m1). For the detection of GFP and human GCSF mRNA, the following primer sequences were used in Real Time PCR assays: GFP: 5′-AGCAGAACACCCCCATCG-3′ and 3′-TCCAGCAGGACCATGTGATC-5′; G-CSF 5′-CTGGCAGCAGATGGAAGAACT-3′ and 3′-CAGGAAGCTCTGCAGATGGGA-5′. The RT-qPCR amplification mixtures contained 20 ηg template cDNA, Taqman Master Mix (10 µL), and probes in a final volume of 20 µL (all from Thermo Scientific). All reactions were run in duplicate on an ABI7500 Sequence Detection System (Thermo Scientific) under standard thermal cycling conditions. The mean Ct (Cycle threshold) values from duplicate measurements were used to calculate expression of the target gene, with normalization to an internal control (*Gapdh*) using the 2^−ΔCt^ formula. Experiments with coefficients of variation greater than 5% were excluded. A non-template control and non-reverse transcription controls were also included. Quantification of parasites by qPCR was performed as previously described (15).

### Statistical Analyses

Statistical comparisons between groups were performed by Student’s *t*-test when comparing two groups and ANOVA followed by Newman–Keuls test for multiple comparisons, using GraphPad Prism program (Software Inc., San Diego, CA, USA) version 5.0. Results were considered significant when *P* < 0.05.

## Results

### G-CSF Overexpression Increases Peripheral Blood Leukocyte Counts and Homing of MSCs to the Hearts of *T. cruzi*-Infected Mice

**Figure 1:**
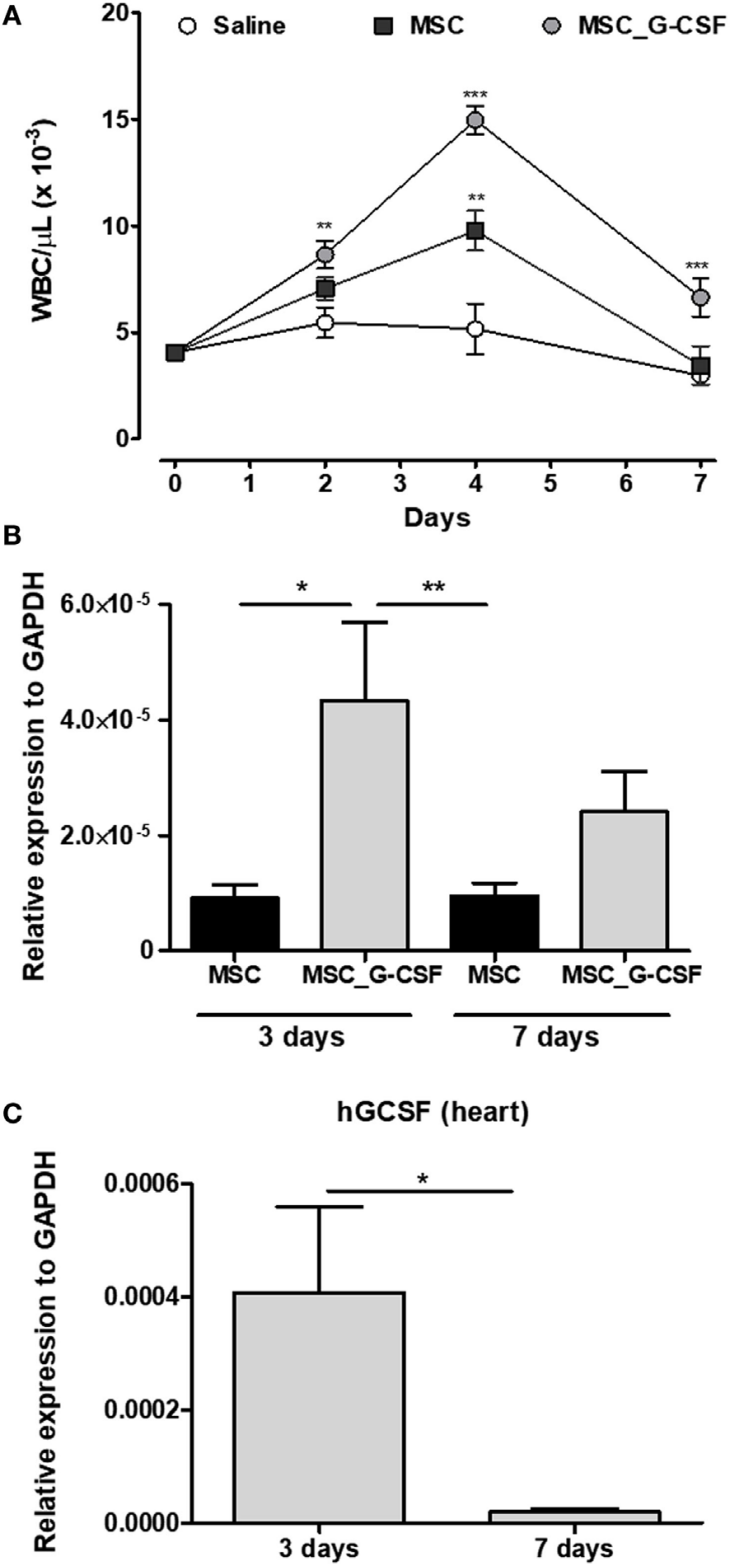
MSC_G-CSF increases peripheral blood leukocyte counts and present enhanced ability to migrate to the heart. **(A)** Naïve mice were i.p. injected with mesenchymal stem cells (MSCs), MSC_G-CSF, or saline. Leukocyte counts were determined before the injection and in the following days. Values represent mean ± SEM of five mice/group. **P < 0.01 and ***P < 0.001 compared to saline-treated mice. **(B,C)** *Trypanosoma cruzi*-infected mice (6 months of infection) were transplanted with one dose of 10^6^ MSCs or MSC_G-CSF. Heart tissue was collected 3 or 7 days later for RT-qPCR analysis of GFP **(B)** or human granulocyte-colony stimulating factor **(C)**. Values represent the mean ± SEM of 4–6 mice/group. *P < 0.05 and **P < 0.01.

In order to establish the therapeutic scheme to be used in our study, we first evaluated the duration of the biological activity of MSC_G-CSF by measuring leukocyte mobilization (Figure S1A in Supplementary Material). A single dose (10^6^ cells, i.p.) of MSCs or MSC_G-CSF was administered to naïve mice and serial hemograms were performed in the following days. As shown in Figure 1A, administration of either wild-type MSCs or MSC_G-CSF increased white blood cell counts, compared to saline injected controls. Transplantation of MSC_G-CSF, however, was associated with even higher numbers of leukocytes, compared to regular MSCs. Leukocyte numbers dropped to the level of controls, in both groups, 7 days after the transplantation (Figure 1A).

In order to evaluate if the cells, administered through intraperitoneal injection, could reach the heart, we performed cell transplantation and tracking in mice chronically infected with *T. cruzi* (Figure S1B in Supplementary Material). Transplanted MSCs and MSC_G-CSF were detected in the hearts of infected mice 3 and 7 days after cell transplantation, as shown by quantification of the reporter gene GFP by RT-qPCR (Figure 1B). In mice transplanted with MSC_G-CSF, however, significantly higher levels of GFP mRNA were detected in the hearts 3 days after transplantation, when compared to mice transplanted with MSCs. We were also able to detect the expression of hG-CSF mRNA in the hearts of MSC_G-CSF-treated mice, 3 days after transplantation. Gene expression of human G-CSF, however, was not sustained, being reduced 7 days after MSC_G-CSF transplantation (Figure 1C). Therefore, the following experiments were performed with repeated cell administrations (Figure S1C in Supplementary Material).

### Mobilization of MDSCs and Tregs After MSC_G-CSF Transplantation

**Figure 2:**
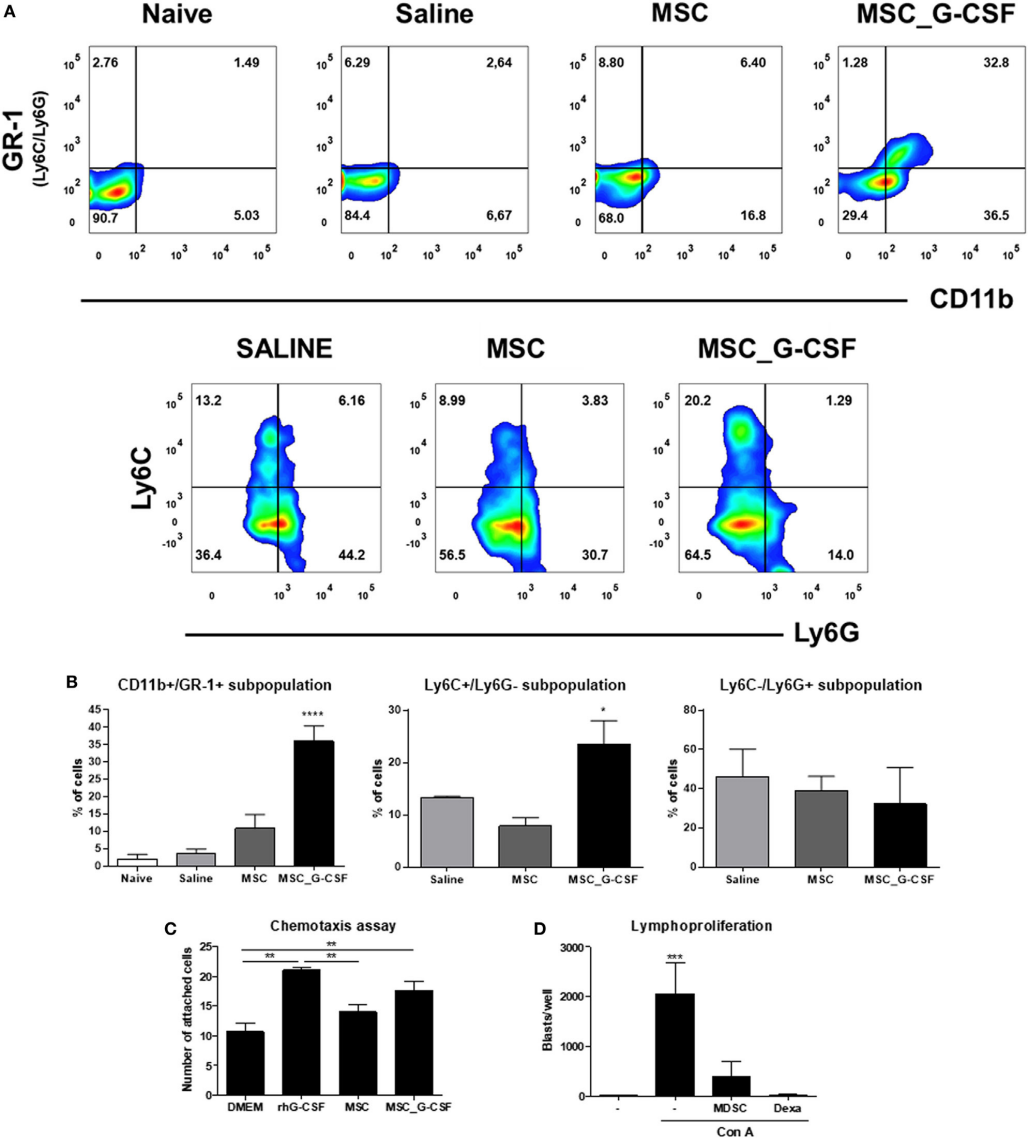
Granulocyte-colony stimulating factor (G-CSF) overexpression in mesenchymal stem cells (MSCs) induces the mobilization of myeloid-derived suppressor cells (MDSCs) to the hearts of *Trypanosoma cruzi*-infected mice. **(A)** Flow cytometry analysis of CD11b/GR-1 and Ly6C and Ly6G subpopulations in the heart tissue of *T. cruzi*-infected mice treated with MSCs, MSC_G-CSF, or saline. **(B)** Percentage of MDSCs (CD11b^+^GR-1^+^) and its subpopulations M-MDSCs (Ly6C^+^Ly6G^−^) and PMN-MDSCs (Ly6C^−^Ly6G^+^) in the hearts of naïve and *T. cruzi*-infected mice from saline, MSCs, or MSC_G-CSF-treated groups. Values represent the mean ± SEM of 3–5 mice/group. **(C)** CD11b^+^GR-1^+^ were sorted from the bone marrow of naïve mice and used in chemotaxis assay in transwell chambers containing Dulbecco’s Modified Eagle’s Medium, human rG-CSF, or conditioned medium of MSCs or MSC_G-CSF. The number of migrating cells was determined 24 h after incubation. Values represent the mean ± SEM of biological replicates. **(D)** Splenocytes from naïve mice were stimulated with concanavalin A in the presence of MDSCs (CD11b^+^GR-1^+^) purified from the hearts of MSC_G-CSF-treated *T. cruzi*-infected mice, or dexamethasone (1 µM) as a positive control, in a 96-well plate. The number of blasts in proliferation was determined using a high content imaging system. Values represent the mean ± SEM of replicates. **P < 0.01; ***P < 0.001.

Since G-CSF is known to induce the mobilization of immune regulatory cells (17), we evaluated the presence of MDSCs and Tregs in the hearts of *T. cruzi*-infected mice 30 days after the beginning of the cell therapy protocol. In order to evaluate the recruitment of MDSCs, hearts of *T. cruzi*-infected mice were digested for analysis of CD11b^+^GR-1^+^, CD11b^+^Ly6C^+^, and CD11b^+^Ly6G^+^ by flow cytometry (Figure 2A). The percentage of MDSCs in both saline and MSCs groups was similar, while MSC_G-CSF significantly increased the percentage of the monocytic subset (M-MDSC) (CD11b^+^Ly6C^+^Ly6G^−^) within the infiltrating CD11b^+^ population (Figure 2B).

In order to evaluate whether MSC_G-CSF-derived secreted factors could induce the recruitment of MDSCs, we performed an *in vitro* chemotaxis assay, using CD11b^+^GR-1^+^ cells sorted from the bone marrow of naïve mice. Conditioned media from MSCs or MSC_G-CSF cultures or human recombinant G-CSF were placed in the lower chamber of transwell plates, while MDSCs were placed in the upper chamber. We found that both MSC_G-CSF conditioned media and regular media supplemented with G-CSF, but not MSCs’ conditioned media, were able to increase the migration of MDSCs (Figure 2C). Finally, in order to demonstrate the suppressor activity of the recruited MDSCs, CD11b^+^GR-1^+^ cells were sorted from the hearts of *T. cruzi*-infected mice treated with MSC_G-CSF and co-cultured with mouse splenocytes activated with concanavalin A (Figure 2D). MDSCs caused a marked reduction in blast formation, showing a potent inhibitory effect on lymphocyte proliferation. As expected, dexamethasone, a positive control, completely blocked blast formation.

**Figure 3:**
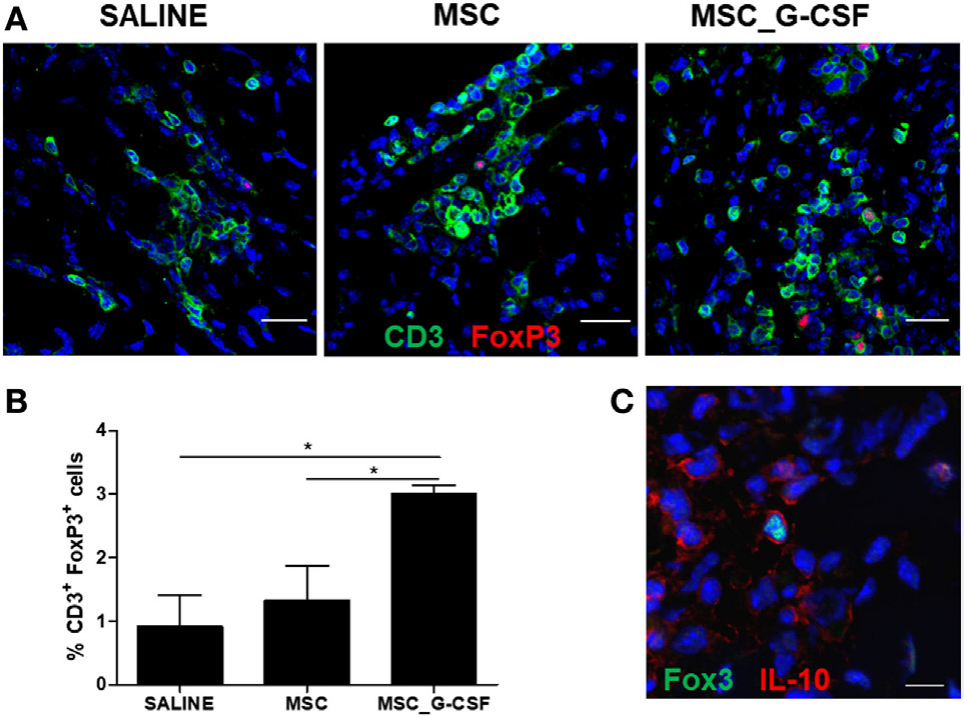
Mobilization of regulatory T cells (Tregs) in the hearts of mice transplanted with MSC_G-CSF. **(A)** Heart sections from *Trypanosoma cruzi*-infected mice treated with mesenchymal stem cells, MSC_G-CSF, or saline were stained for CD3 (green) and FoxP3 (red). **(B)** Quantification of Tregs represented the percentage of CD3^+^FoxP3^+^ within CD3^+^ T cells. Values represent the mean ± SEM of five mice/group. *P < 0.05. **(C)** Heart section from *T. cruzi*-infected mice treated with MSC_G-CSF co-stained for FoxP3 (green) and IL-10 (red). Bars = 50 µm **(A)** and 10 µm **(C)**.

Next, we evaluated the frequency of Tregs in the hearts, by performing immunostaining for CD3 and FoxP3 in heart sections of mice treated with saline, MSCs, or MSC_G-CSF (Figure 3A). Quantification showed a significant increase (~threefold) in the percentage of Tregs, compared to saline and MSCs groups (Figure 3B). Confocal analysis showed that FoxP3^+^ cells, in addition to other cell populations, were positive for IL-10 (Figure 3C).

### Improvement of Exercise Capacity and Reduction of Inflammation and Fibrosis in Mice Transplanted With MSC_G-CSF

**Figure 4:**
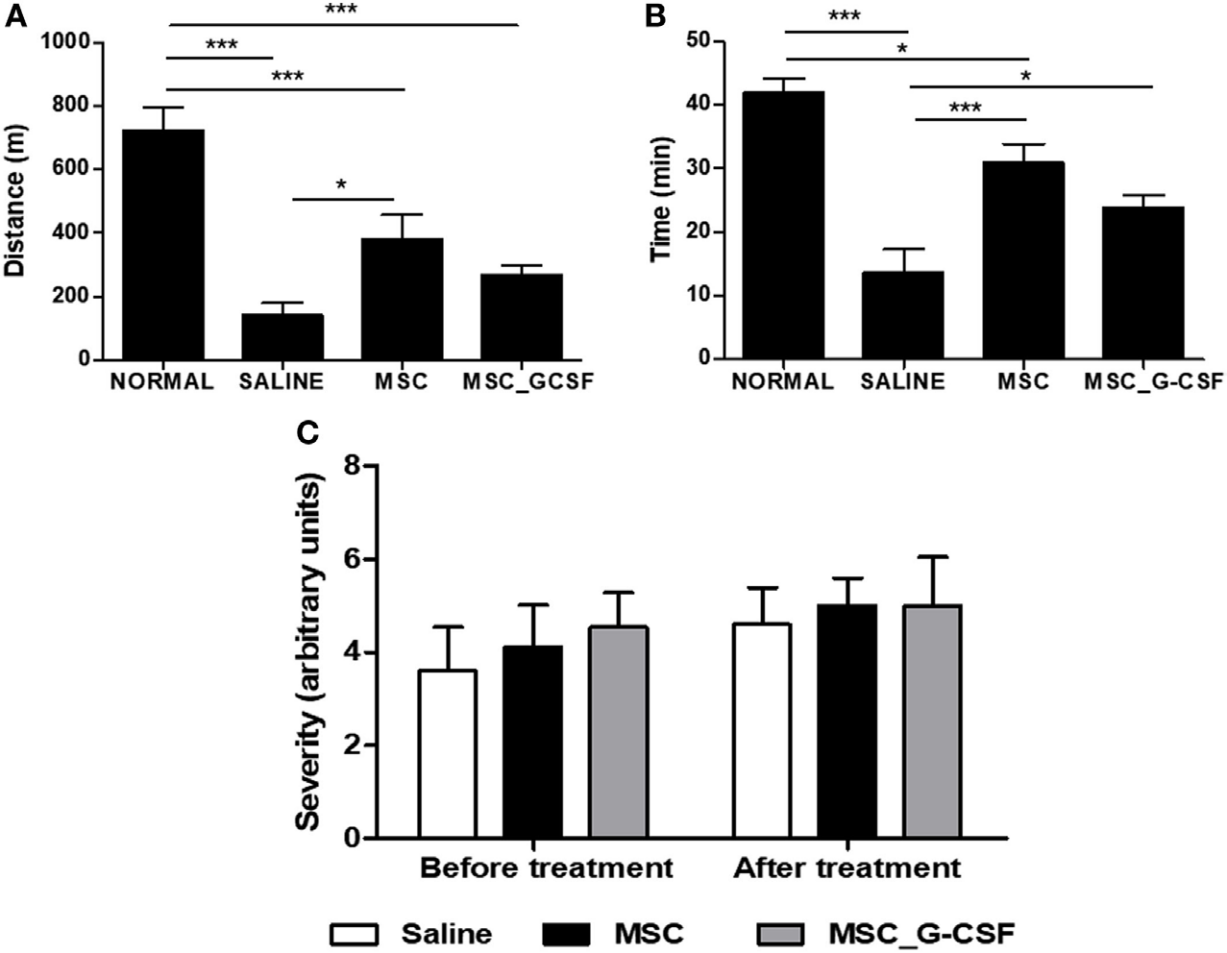
Functional analyses of mice treated with mesenchymal stem cells (MSCs) or MSC_G-CSF. Naïve and *Trypanosoma cruzi*-infected mice (6 months of infection) were evaluated by exercise capacity and electrocardiogram before and 60 days after the beginning of treatment with MSCs, MSC_G-CSF, or saline. Distance **(A)** and time **(B)** of exercise evaluated in a treadmill test. Values represent the mean ± SEM of 7–9 mice/group. **(C)** Conduction disturbances evaluated by electrocardiography. Severity was scored as follows: 0, no cardiac conduction disturbances; 1, first-degree atrium-ventricular block; 2, intraatrium conduction disturbance; 3, junctional rhythm; 4, intraventricular conduction disturbance; 5, ventricular bigeminism; 6, polymorphic ventricular tachycardia; and 7, AV dissociation. Values represent the mean ± SEM of 9–10 mice/group. *P < 0.05; ***P < 0.001.

In order to evaluate long-term effects of the cell therapy and assess functional recovery, treadmill tests and electrocardiograms were performed before treatment and 60 days after the beginning of the cell therapy protocol. Exercise capacity, evaluated by a treadmill test, showed a significant reduction in distance and time run in saline-treated, *T. cruzi*-infected mice, compared to naïve controls (Figures 4A,B). Both MSCs and MSC_G-CSF treated mice showed a partial recovery in exercise capacity. ECG analyses were also performed before and after treatment. All *T. cruzi*-infected groups presented similar degrees of conduction disturbances, which included first-degree atrium-ventricular block, intra-atrial conduction disturbance, junctional rhythm, intraventricular conduction disturbance, ventricular bigeminism, polymorphic ventricular tachycardia, and atrioventricular dissociation (Figure 4C). These findings were not reversed by any of the cell therapy protocols evaluated.

**Figure 5:**
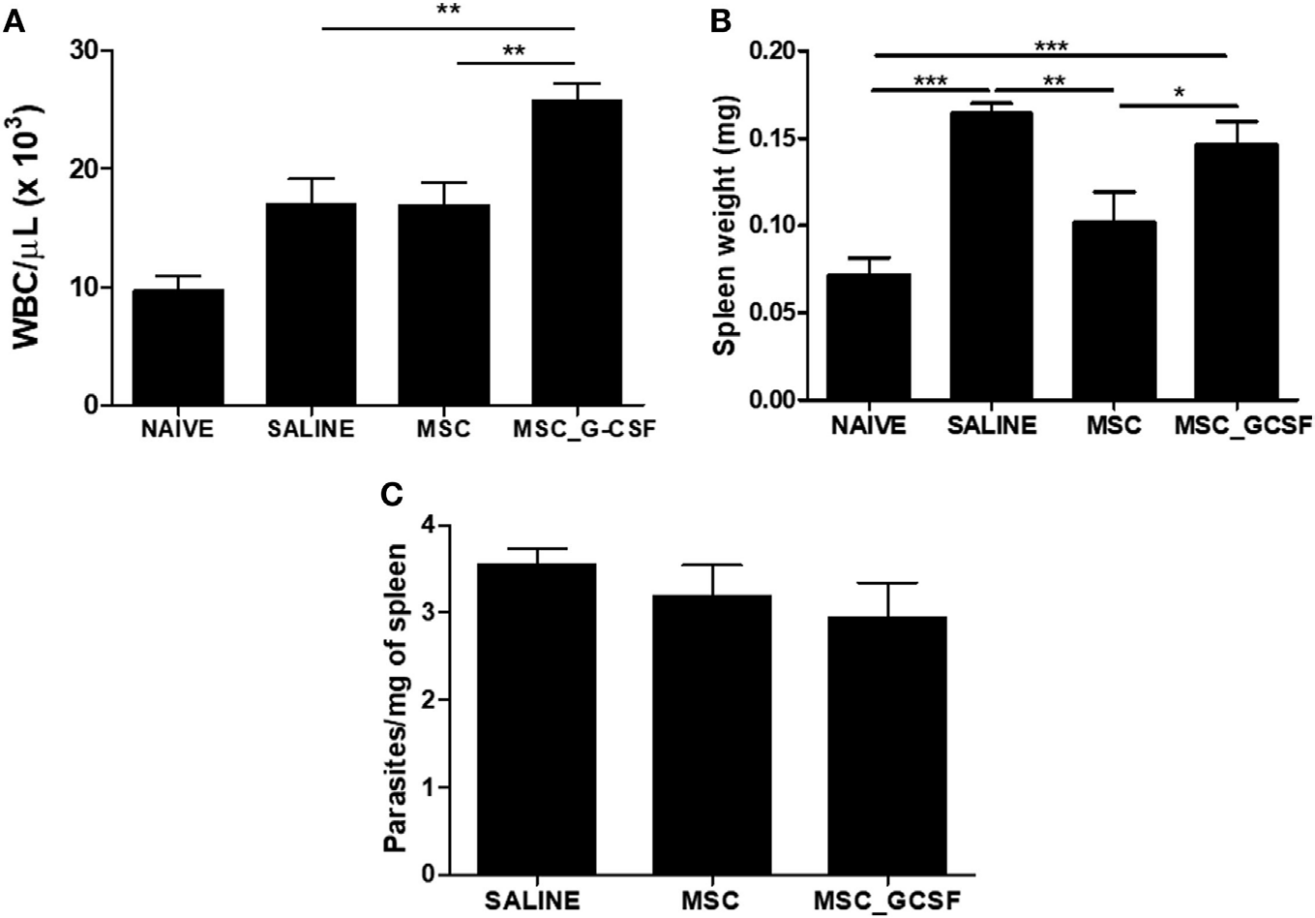
Effects of cell therapy on leukocyte mobilization, spleen weight, and parasitism. **(A)** Peripheral blood leukocyte counts 60 days after the treatment with mesenchymal stem cells (MSCs), MSC_G-CSF, or saline. **(B)** Spleen weight was found to be increased in infected mice, and decreased by treatment with MSCs, but not MSC_G-CSF. **(C)** Parasitism in the spleens, evaluated by qPCR, was not altered by the cell therapy. Values represent the mean ± SEM of 7–9 mice/group. *P < 0.05; **P < 0.01; ***P < 0.001.

In accordance with the results of the 7 days-time point, peripheral blood leukocyte counts were found to be significantly increased in the MSC_G-CSF group, compared to the other groups, 60 days after the beginning of the treatment (Figure 5A). Moreover, spleen weight, which was increased by infection in saline-treated mice, compared to naïve controls, was reduced in MSCs group, but not in MSC_G-CSF group (Figure 5B). In order to determine if the cell therapy affected the parasite load, we performed RT-qPCR to quantify *T. cruzi* parasites in the spleen. We found that infected mice treated with either MSCs or MSC_G-CSF had similar parasite load than saline-treated mice (Figure 5C).

**Figure 6:**
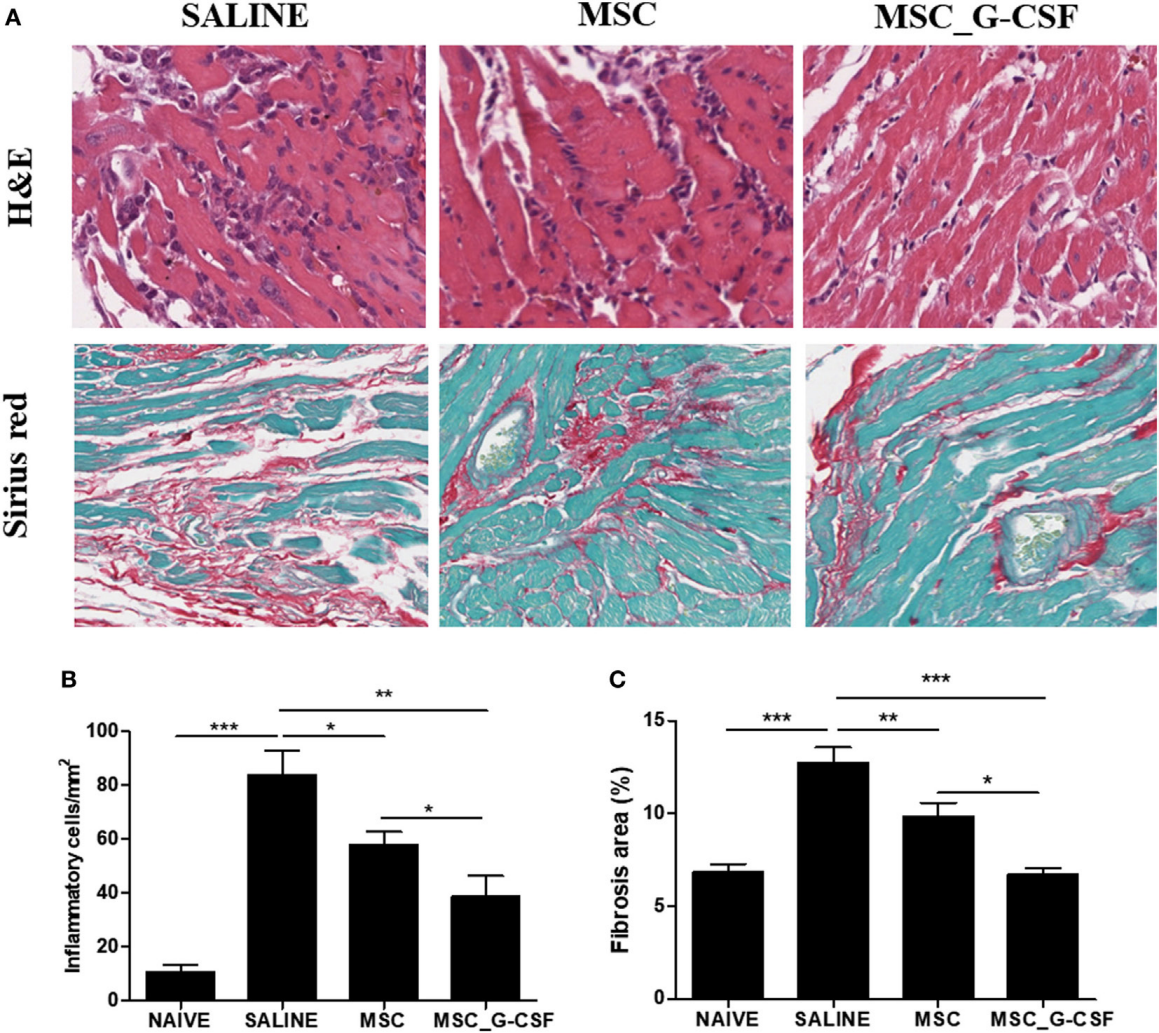
Reduction of heart inflammation and fibrosis after treatment with mesenchymal stem cells (MSCs). Hearts of naïve and *Trypanosoma cruzi*-infected mice treated with MSCs, MSC_G-CSF, or saline were evaluated 60 days after the beginning of treatment. **(A)** Heart sections of saline, MSCs, and MSC_G-CSF-treated mice stained with hematoxylin and eosin (H&E) or Sirius red. Infiltrating inflammatory cells **(B)** and fibrosis area **(C)** were quantified in heart sections by morphometrical analysis in sections stained with H&E or Sirius red, respectively. Values represent the mean ± SEM of 7–9 mice/group. *P < 0.05; **P < 0.01; ***P < 0.001. Magnification = 400× for H&E images and 200× for Sirius red images.

Heart sections were prepared from mice euthanized 60 days after beginning of treatment and stained with H&E and Sirius red to evaluate inflammation and fibrosis, respectively (Figure 6A). A significant reduction of inflammation and fibrosis was observed in the hearts of mice treated with either MSCs or MSC_G-CSF, compared to saline (Figures 6B,C). In the group treated with MSC_G-CSF, however, a more pronounced reduction in the number of infiltrating inflammatory cells and in the area of fibrosis was observed (Figure 6C).

**Figure 7:**
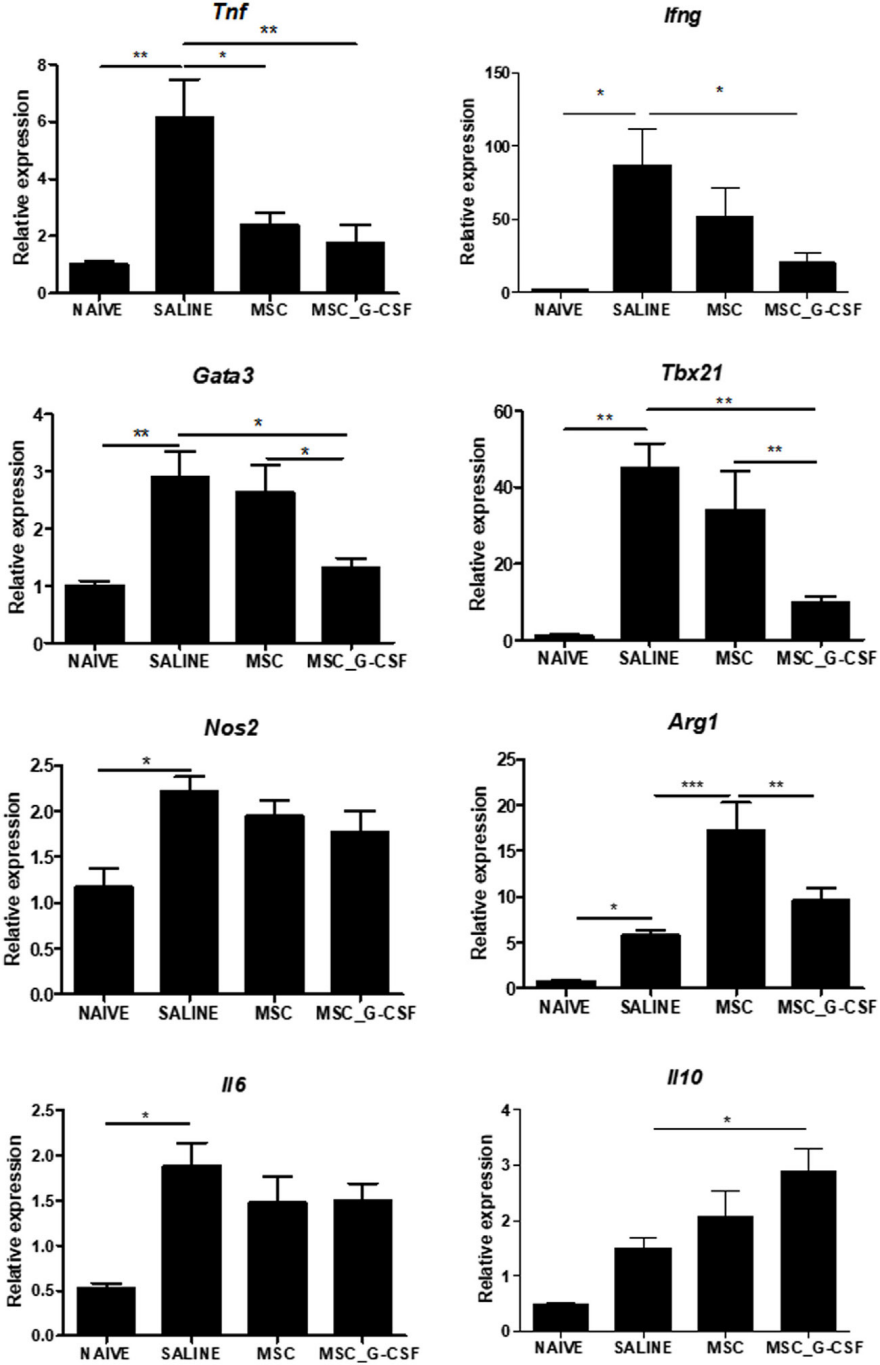
Modulation of gene expression for inflammatory mediators in the hearts of mice transplanted with MSC_G-CSF. Hearts from naïve and *Trypanosoma cruzi*-infected mice treated with mesenchymal stem cells, MSC_G-CSF, or saline were submitted to RT-qPCR analysis for evaluation of the gene expression for TNFα, IFNγ, GATA3, Tbet, iNOS, Arg, IL-6, and IL-10. Results are shown as relative expression to GAPDH. Values represent the mean ± SEM of 5–7 mice/group. *P < 0.05; **P < 0.01; ***P < 0.001.

Gene expression analysis was performed in heart samples obtained from *T. cruzi* infected, in order to evaluate the expression levels of transcription factors and mediators associated with the immune response and inflammation. We found that infected mice presented a significant increase in inflammatory mediators, including inflammatory cytokines TNFα, IL-6, IFNγ, and transcription factors associated with Th1 and Th2, respectively, Tbet (Tbx21) and GATA-3, when compared to naïve controls (Figure 7). A significant reduction in the expression levels of TNFα, IFNγ, GATA3, and Tbet was seen in MSC_G-CSF mice, while the expression of the anti-inflammatory cytokine IL-10 was significantly increased, compared to saline group. In contrast, treatment with MSCs caused a significant reduction in TNFα, while increasing arginase 1 expression (Figure 7).

## Discussion

The use of cell therapies has been investigated in a number of cardiac diseases, including chronic Chagas disease, but currently no efficient treatment has been established for clinical practice (18, 19). Previous studies have shown improvements after MSC-based therapy in the mouse model of Chagas disease (11–14, 20). Genetic modification of MSCs is a promising strategy to deliver therapeutic agents for the treatment of several medical applications, which is already progressing into clinical trials (5). MSCs producing anti-inflammatory cytokines, such as IL-10 and TGF-β, have been tested in autoimmune disease models (21, 22). Here we investigated the effectiveness of combined gene and cell therapy in a mouse model of chronic Chagas disease. We found that G-CSF-overexpressing MSCs have increased immunomodulatory properties, promoting a more profound reduction in inflammation, fibrosis, and modulation of inflammatory mediators, when compared to regular MSCs. This effect was associated with an increased ability to recruit suppressor cell populations, such as Tregs and MDSCs.

The interplay between G-CSF-overexpressing MSCs and suppressor cells explains the increased immunomodulatory potential observed *in vivo*, since MSC_G-CSF present similar ability to suppress lymphocyte proliferation *in vitro*, when compared to wild-type MSCs (16). G-CSF was previously shown to promote mobilization of Tregs, modulating allogeneic immune responses (23). Moreover, treatment with G-CSF recruits Tregs to the hearts of mice chronically infected with *T. cruzi* (14). In line with this, we found here a higher frequency of Tregs in the hearts of chagasic mice transplanted with MSC_G-CSF. Treg cells were shown to act ameliorating cardiac damage and suppressing cardiac hypertrophy and fibrosis in a model of angiotensin II-mediated cardiac damage (24).

A marked increase in MDSCs was also found in the hearts of MSC_G-CSF-treated mice. These cells play important immune regulatory roles in several diseases, including chronic infections, autoimmunity, and cancer (9). G-CSF was found to be a key molecule in the regulation of migration, proliferation, and function maintenance of MDSCs in a mouse model of colitis (25). In fact, by using a chemotaxis assay, we found that culture supernatants from MSC_G-CSF, but not MSCs, had similar MDSCs recruitment capacity than the recombinant G-CSF. A previous study showed the increase of MDSCs in spleens and hearts during acute *T. cruzi* infection in mice (26). These cells express Arg-1 and iNOS, which suppress T cell proliferation, allowing the host to avoid an excessive immune response that may also cause damage (27). In the present study, accumulation of MDSCs was associated with decreased inflammation, without disrupting the parasitism control by the anti-*T. cruzi* immune response.

Myeloid-derived suppressor cells extracted from the hearts were also shown to be functional and to suppress T cell activation *in vitro*. It is known that MDSCs are potent suppressor cells that act through the secretion of anti-inflammatory cytokines, such as IL-10 and TGF-β. Moreover, both M- and PMN-MDSCs have been recently shown to exert antihypertrophic and anti-inflammatory properties, in the context of heart failure, through IL-10 (28). A marked increase in IL-10 expression was observed in the hearts of mice treated with MSC_G-CSF in our study.

A number of studies have shown that G-CSF can induce T cell tolerance (29). In addition to its indirect effects *via* mobilization of suppressor cell populations, G-CSF may exert a direct effect on T cells. The interaction between G-CSF and its receptor (G-CSFR), which is expressed in various hematopoietic cell types, including T cells, can favor Th2 over Th1 imbalance by the induction of activation of GATA3 (30). The analysis of GATA3 and Tbet expression, the transcription factors associated with Th2 and Th1 differentiation, respectively, in the hearts of MSC_G-CSF mice failed to show a polarization to Th2 phenotype, since the gene expression of both transcription factors were reduced compared to saline-treated chagasic control mice.

A role for Tregs in the control of pathological T cell responses in chronic Chagas disease cardiomyopathy has been suggested by studies showing that patients with the indeterminate form of the disease (asymptomatic) have increased frequency of Tregs in the peripheral blood, compared to patients with severe cardiomyopathy (29, 30). In contrast, there are no studies indicating a protective role for MDSCs in chronic Chagas disease patients. Our results demonstrate that MDSCs, with confirmed immunosuppressive actions, accumulate in the hearts of MSC_G-CSF-treated mice, being associated with reduction of cardiac inflammation. This suggests that strategies to increase MDSC mobilization may be beneficial for the treatment of patients with chronic Chagas disease cardiomyopathy.

The presence of multifocal inflammatory infiltrates, composed mainly of mononuclear cells, is a hallmark of chronic Chagas disease cardiomyopathy. Here we show that G-CSF-overexpressing MSCs reduce not only the number of inflammatory cells but also the production of pro-inflammatory cytokines, such as TNFα and IFNγ. These are important mediators of chronic Chagas disease cardiomyopathy, as shown in the mouse model (10, 31) and in the human disease (32–34). This reduction in pro-inflammatory mediators correlated with an increase in IL-10, a potent anti-inflammatory cytokine.

Also, MSC_G-CSF therapy caused a more pronounced reduction in cardiac fibrosis than wild-type MSCs. The finding of reduced cardiac fibrosis may be the result of decreased cardiac inflammation, since it is known that the exacerbated immune response, frequently seen in Chagas disease, progressively leads to the destruction of cardiac myocytes, increasing fibrosis. Cardiac fibrosis may promote conduction disturbances, leading to arrhythmias and accounting for a high percentage of the mortality due to chronic Chagas disease (35). Despite the reduction in fibrosis, treatment with MSC_G-CSF did not improve the electrical disturbances. Mice treated with stem cells, however, had a partial recovery in the exercise capacity. This suggests that cell therapy may be more effective if applied earlier to prevent or block the evolution of the disease.

Immunosuppressive drugs can reactivate infection in mice and patients with residual parasitism during the chronic phase of infection (36, 37). To rule out that the immunosuppressive activity of MSC_G-CSF were reactivating the parasite growth, we performed RT-qPCR analysis to quantify *T. cruzi* parasite load in spleen samples from infected mice. This analysis demonstrated that, despite the marked immunosuppressive activity of MSC_G-CSF, the cell transplantation scheme did not interfere with host–parasite equilibrium. Since the elimination of the parasite is desirable, the possibility that combining MSC_G-CSF therapy with benznidazole administration promotes additional improvements than the cell therapy alone needs to be further investigated.

While the use of many recombinant factors is frequently associated with side effects, the use of recombinant G-CSF is considered safe and well tolerated. Moreover, we have previously shown that recombinant G-CSF promotes beneficial effects in the mouse model of Chagas disease (14). We hypothesized here that MSC_G-CSF could offer some advantages, such as the ability to migrate to the injury site and promote local release of G-CSF and other growth factors which may act synergistically to promote tissue repair and immunomodulation. Our previous results with recombinant G-CSF, however, were effective not only in reducing inflammation and fibrosis, as seen with MSC_G-CSF but also caused reduction of tissue parasitism and amelioration of arrythmias. Although the effect of MSC_G-CSF may not bring advantage when compared to repeated courses of G-CSF alone, we have here demonstrated, for the first time, that genetic manipulation may improve the therapeutic effects of MSC in the context of Chagas disease. The fact that transplantation of MSC alone causes some immunomodulatory action suggests that the effect seen in mice treated with MSC_G-CSF is due to the release of G-CSF and other mediators.

In conclusion, we show that overexpression of G-CSF in MSCs potentiates their *in vivo* immunosuppressive effects in a model of chronic Chagas disease. Moreover, our results indicate a role of MDSCs in the regulation of pathological immune responses, opening new avenues for the development of cell-based therapies in chronic Chagas disease.

## Ethics Statement

All procedures described had prior approval from the local animal ethics committee under number 012/09 (FIOCRUZ, Bahia, Brazil).

## Author Contributions

DS, JV, CA, CV, BP, VR, GC, PD, SM, and CN performed the experiments. DS, BS, JV, VR, and BP analyzed the data. DS, BS, RR-d-S, and MS conceived the study and wrote the manuscript.

## Conflict of Interest Statement

The authors declare that the research was conducted in the absence of any commercial or financial relationships that could be construed as a potential conflict of interest. The reviewer FA declared a shared affiliation, though no other collaboration, with several of the authors DS, BS, JV, CA, VR, CN, and MS to the handling Editor.

## Supplementary Material

Figure S1 Experimental design. **(A)** Establishment of dose and administration regimen. C57BL/6 mice were treated with a single dose of 10^6^ mesenchymal stem cells (MSCs) or MSC_G-CSF (i.p) and serial hemograms were performed during 7 days. **(B)** Cell tracking was performed in C57BL/6 mice chronically infected with *T. cruzi* (6 months after infection) 3 and 7 days after administration of 10^6^ MSCs or MSC_G-CSF (i.p), for detection of GFP^+^ cells and human granulocyte-colony stimulating factor gene in the heart. **(C)** C57BL/6 infected with *T. cruzi* was treated 6 months after infection with 10^6^ MSCs or MSC-GCSF, every 7 days, during 60 days. Saline-treated and naïve mice were used as controls. Mice were euthanized for 30 days after the first administration of cells for evaluation of recruitment of myeloid-derived suppressor cells and regulatory T cells to the heart.
