# Supplementary figures and images for: Granulocyte-Colony Stimulating Factor-Overexpressing Mesenchymal Stem Cells Exhibit Enhanced Immunomodulatory Actions Through the Recruitment of Suppressor Cells in Experimental Chagas Disease Cardiomyopathy
Source: Front Immunol. 2018 Jun 25;9:1449. doi: 10.3389/fimmu.2018.01449 (PMC6036245; doi:10.3389/fimmu.2018.01449)

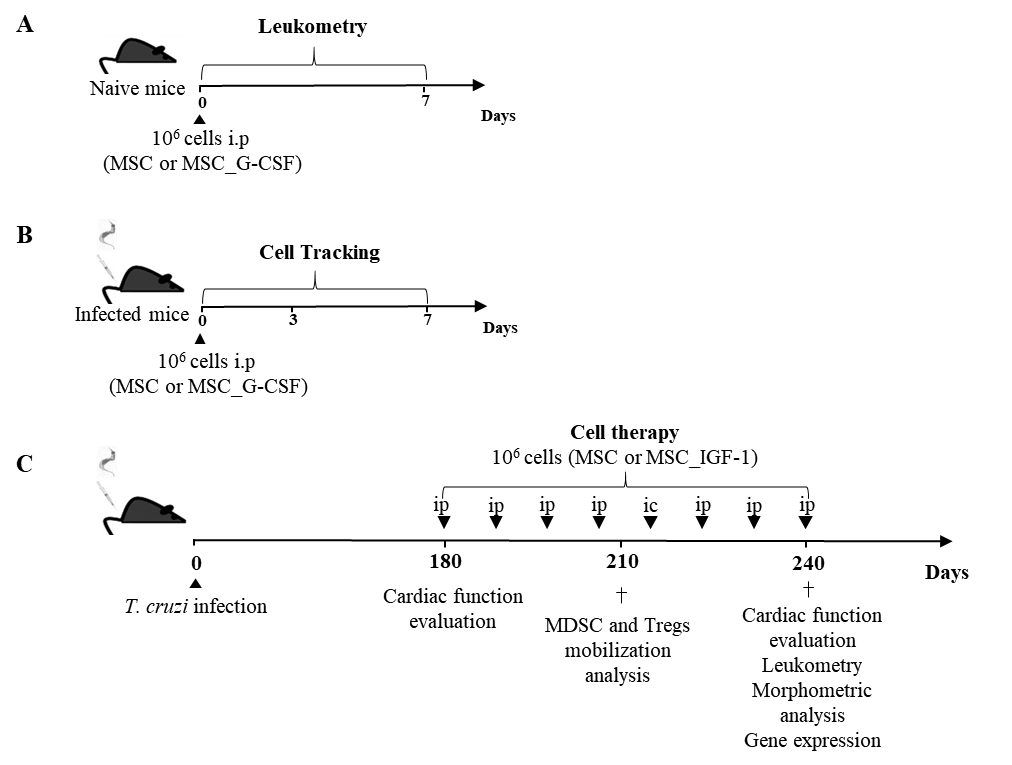

Supplement: Figure S1 — Experimental design. (A) Establishment of dose and administration regimen. C57BL/6 mice were treated with a single dose of 106 mesenchymal stem cells (MSCs) or MSC_G-CSF (i.p) and serial hemograms were performed during 7 days. (B) Cell tracking was performed in C57BL/6 mice chronically infected with T. cruzi (6 months after infection) 3 and 7 days after administration of 106 MSCs or MSC_G-CSF (i.p), for detection of GFP+ cells and human granulocyte-colony stimulating factor gene in the heart. (C) C57BL/6 infected with T. cruzi was treated 6 months after infection with 106 MSCs or MSC-GCSF, every 7 days, during 60 days. Saline-treated and naïve mice were used as controls. Mice were euthanized for 30 days after the first administration of cells for evaluation of recruitment of myeloid-derived suppressor cells and regulatory T cells to the heart. [file image_1.tiff]
